# Controlled growth of citrate-stabilized gold nanoparticles using a semi-continuous seed-mediated route

**DOI:** 10.1186/s11671-025-04189-8

**Published:** 2025-02-17

**Authors:** Muhammad Bilal, Sulalit Bandyopadhyay

**Affiliations:** Particle Engineering Centre, Department of Chemical Engineering, Norwegian University of Science and Technology, 7491 Trondheim, Norway

## Abstract

Gold nanoparticles (Au NPs) hold unique optical and electronic properties due to their surface plasmon resonance. The size, shape, and surface chemistry of Au NPs are key parameters for altering their optical properties to fine-tune Au NPs for specific applications. We report a size-controlled synthesis of citrate-stabilized Au NPs via the Turkevich method, followed by their growth through a semi-continuous seed-mediated approach. Au NPs of up to 53 nm were synthesized by controlled addition of chloroauric acid (HAuCl₄) to pre-prepared citrate stabilized Au NP seeds. Our approach leverages the residual sodium citrate from the Turkevich reaction to reduce HAuCl₄ during seed-mediated growth of Au NPs. Notably, we observed that a boiling temperature, as opposed to 70 °C, provided better control over nanoparticle size and morphology. Our method addresses several challenges associated with seed-mediated growth by yielding relatively spherical, monodisperse, citrate-stabilized, water-dispersible Au NPs in a single growth step, without sacrificing yield. Furthermore, a kinetics study revealed a continuous increase in particle diameter over the reaction period, hinting at the continuous and uniform growth of Au NPs.

**Supplementary Information:**

The online version contains supplementary material available at 10.1186/s11671-025-04189-8.

## Introduction

In recent years, gold nanoparticles (Au NPs) have attracted significant attention due to their unique optical properties stemming from their localized surface plasmon resonance (LSPR) which can be controlled by manipulating their sizes and shapes [1]. Such NPs find applications, particularly in nanomedicine, including biosensing, bioimaging, and targeted drug delivery among others. A simple and robust synthesis method capable of meeting the desired physicochemical properties of Au NPs is therefore warranted.

The Turkevich method is one such approach largely used for synthesizing spherical Au NPs in the range of 10–30 nm, due to its simplicity and reproducibility. The particle size can be controlled by altering the molar ratio of chloroauric acid (gold precursor) to trisodium citrate (reducing agent), temperature, pH, and order of reagent addition, etc. [2]. An inherent challenge of the classical Turkevich method is the synthesis of larger Au NPs (> 30 nm) with low polydispersity and high sphericity. Such Au NPs, owing to their high scattering cross-section, are ideal for imaging applications [3], enhancing contrast and visibility in techniques such as dark-field microscopy [4] and optical coherence tomography [5]. Furthermore, their large surface area makes them ideal for Surface-Enhanced Raman Scattering (SERS) providing enhanced sensitivity, whereas their SPR shifting to longer wavelengths renders them ideal for biosensing and bioimaging applications [6].

To overcome the limitations of the Turkevich method, seed-mediated growth techniques have been employed, which rely on heterogeneous nucleation. These start with the formation of gold seeds that act as nucleation sites for particle growth, which happens by the autocatalytic growth of metal atoms onto the surface of seeds. By separating nucleation and growth steps, it is possible to obtain Au NPs of uniform and narrow-size distributions, [7] which can be controlled by adjusting reaction conditions such as the concentration of the gold precursor, seed solution, reducing agent, temperature, capping agents and so on [8, 9]. Jana et al. proposed a method for particles sized between 5 and 40 nm using cetyl-trimethylammonium bromide (CTAB) as the capping agent and sodium borohydride as the reducing agent [10]. However, the presence of CTAB on the surface of Au NPs complicates functionalization with biological ligands crucial for biomedical applications [11]. Contrarily, there are aqueous chemistry-based methods that do not employ surfactants—one such method has been reported by Bastus et al. that takes three growth steps to attain a size of ∼ 30 nm using sodium citrate as reducing as well as capping agent (although larger sizes can be achieved with more number of growth steps) [12]. The process requires a highly concentrated ${\text{HAuCl}}_{4}$ solution (∼ 60 mM) which makes it resource-intensive and takes multiple steps to achieve the desired size making it a time-consuming process. On the other hand, the process reported by Zeigler et al. uses a combination of ascorbic acid and trisodium citrate as reducing and stabilizing agents but has a significantly low yield due to the dilution of seed solution over multiple growth steps [13].

In another seeded growth approach, Andalibi et al. reported a fast injection of the gold precursor into a seed solution containing added sodium citrate(reductant), leading to, a wider size distribution due to homogeneous nucleation of new Au NPs [14]. Similarly, Brown et al. reported a widely used method for synthesizing Au NPs via a seeded approach using sodium citrate and hydroxylamine as reductants, the method, however, leads to the formation of non-spherical shapes such as elliptical, rods, and cubic particles after a few subsequent growth steps [15]. Furthermore, CTAB, ascorbic acid, and sodium citrate were used in a seeded method reported by Rodriguez-Fernandez et al. in which, they purified the solution after the first growth step to isolate spherical nanoparticles. Unlike the study of Jana et al. [10], they used the supernatant containing spherical particles for subsequent growth steps [16]. Due to the use of several reactants and cleaning processes in between, this method may be rendered time-consuming and resource-intensive besides giving rise to broader size distribution owing to a fast injection of precursor.

While most of these variations may be possible to achieve in batch processes, a semi-continuous process is seen as an important transition from lab scale to industrial scale. However, in such a setting, a slow introduction of gold precursor is required to keep the supersaturation low enough not to cross the nucleation barrier but favor Au NPs’ growth solely. From the plethora of research, some of which was summarized above, we know that the processes of seeded growth are typically accompanied by nucleation of new particles. This nucleation can occur either homogeneously or in close proximity to the existing seed surface, a phenomenon known as true catalytic secondary nucleation, or near-surface nucleation, followed by particle-mediated growth. Moreover, fast injection of the gold precursor can result in a broader size distribution, primarily due to the homogeneous nucleation of new Au NPs. Addressing these challenges in the field, herein, we propose a one-step seed-mediated growth method carried out in a semi-continuous fashion featuring slow addition of the gold precursor to avoid homogeneous nucleation for controlled synthesis of Au NPs. Our approach aims to address some of the important shortcomings from previous reports, such as ease of post-production modification, low yield, time-consuming process, and a wider size distribution of Au NPs among others. Our method has the potential to produce relatively spherical Au NPs of the desired size ranging between 21 and 53 nm in a single growth step without compromising the yield.

## Experimental section

### Materials

HAuCl_4_ · 3H_2_O (≥ 99.9%), sodium citrate dihydrate (≥ 99%) and nitric acid (90%) were purchased from Sigma Aldrich. Aladdin SyringeONE Programmable Syringe Pump AL-1000 from World Precision Instruments was used for pumping the gold precursor solution during the reactions. Milli-Q (MQ) water, with a resistivity of 18.2 MΩcm, obtained from Sartorius Arium Mini Plus, was used in all the experiments for solution preparation, cleaning, and characterization of the synthesized NPs.

### Methods

### Synthesis of gold nanoparticles

This method was adapted and modified from the one reported by Turkevich et al. [17]. Briefly, in a round-bottom flask equipped with a condenser, 199 mL of a 0.25 mM HAuCl_4_ solution was added. A clean magnetic stirrer was introduced into the round-bottom flask, and the stirring speed was adjusted to 480 rpm. The solution was then subjected to reflux at 125 °C until a vigorous boil was achieved. The reaction temperature of 125 °C corresponds to the oil bath temperature monitored and controlled using a thermostat. This is important as the rate of Au NP synthesis is accelerated at higher temperatures, and has been shown to improve the monodispersity of the NPs [2]. At the onset of boiling, a one-milliliter aliquot of a freshly prepared 500 mM sodium citrate solution was swiftly added, resulting in a final sodium citrate concentration of 2.5 mM. The reflux was continued for 15 min, and the reaction mixture turned into a distinctive ruby-red coloration. The as synthesized Au NPs were used immediately or stored at 4 °C in a glass vial and used as seeds in the next step, without further purification.

### Seed-mediated growth of gold nanoparticles

The following method has been modified from a study conducted by Bastus et al. [12]. For the seed-mediated growth of Au NPs, 10 mL of previously synthesized Au NP seed solution was transferred into a two-necked (100 mL) round-bottom flask, securely positioned in an oil bath on a heating plate. A clean magnetic stirrer was introduced into the round-bottom flask, and the stirring speed was adjusted to 320 rpm. The solution was heated to 125 °C under reflux. The reaction temperature of 125 °C corresponds to the oil bath temperature monitored and controlled using a thermostat. A syringe containing 10 mL of a 0.25–1.0 mM of HAuCl_4_ concentration, was used to feed the solution into the round-bottom flask with the help of a syringe pump (World Precision Instruments AL-1000, flow rate being controlled in the range 335–670 µL/min. The exact concentrations and flow rates of gold precursor for all experiments are given in Table 1 of supporting information (SI). The pumping of HAuCl_4_ commenced when the reaction temperature reached 125 °C, and upon the completion of the transfer of the gold precursor, the pump was switched off. Subsequently, heating was discontinued, and the round bottom flask was carefully removed from the oil bath, allowing it to cool down while maintaining stirring. The resulting Au NP solution in the round bottom flask was then transferred to a 50 mL glass vial and stored at 4 °C without further cleaning.

### Characterization techniques

The Au NPs were imaged using a Hitachi High-Tech SU9000 scanning transmission electron microscope (S(T)EM). For sample preparation, approximately 100 µL of the Au NP solution was deposited onto a Formavar-coated 300 mesh copper grid, obtained from Electron Microscopy Sciences. Excess liquid was carefully removed with a Kimtech wipe, and the grids were allowed to dry before imaging. Size distribution measurements were performed by analyzing at least 200 nanoparticles using ImageJ software. The sizes were measured using ImageJ software's threshold function, while the aspect ratio of elongated nanoparticles was determined by measuring the longest and shortest axes.

The localized surface plasmon resonance (LSPR) properties of the Au NPs were analyzed using an Agilent Cary 60 UV–Vis spectrophotometer. One milliliter of the as-prepared colloidal Au NP solution was placed in a disposable cuvette. Spectral measurements were conducted over a wavelength range of 200–800 nm at room temperature, with a scan resolution of 1 nm.

The gold content in the Au NP samples and control solutions was quantified using an Agilent MP-AES 4210 Optical Emission Spectrometer. To isolate unreacted gold ions in the Au NP samples, the nanoparticles were centrifuged at 14,500 rpm for 20 min, and the supernatant was collected for analysis. For the controls, corresponding concentrations of HAuCl₄ were used to establish Au ions in precursor solutions. Prior to analysis, all samples were digested using a Berghof Speedwave XPERT microwave digestion system by diluting them in 65% nitric acid to ensure complete dissolution of gold species. The digested samples were analyzed in triplicates using MP-AES, and the average value for each sample was calculated, along with the corresponding standard deviation, to ensure precision and reliability.

## Results and discussion

We explore in this work the effect of reaction conditions in the seed-mediated growth method for controlling the size, size distribution, and morphology of the resultant Au NPs, the initial seeds being synthesized by the well-established Turkevich method.

### Synthesis of Au NPs via the Turkevich method

**Fig. 1:**
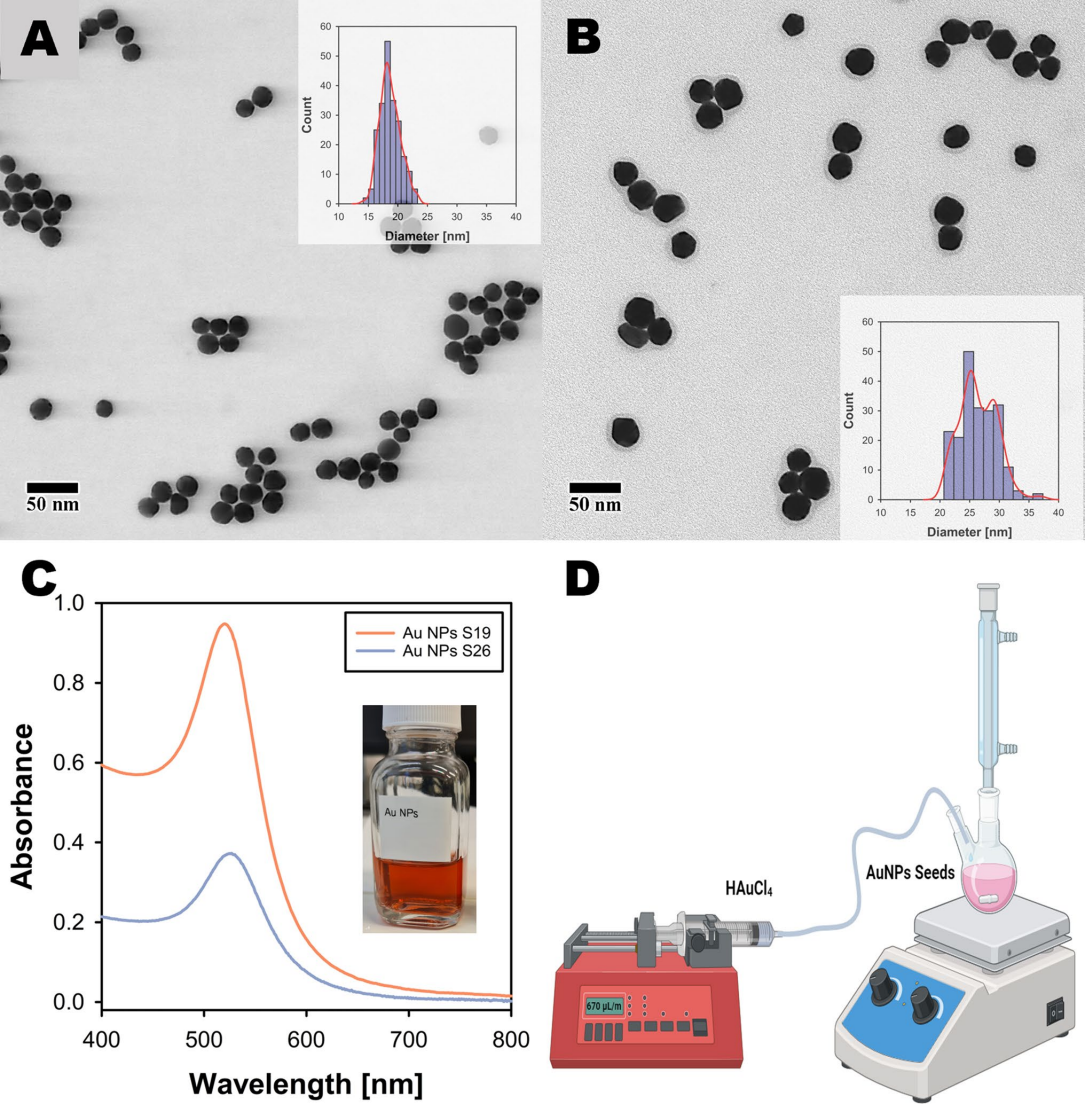
The Turkevich gold nanoparticles at various chloroauric acid concentrations. **A**–**B** shows representative S(T)EM images of Au NPs synthesized from 0.25 and 0.125 mM of HAuCl₄, with insets showing particle size distributions measured using Image J. **C** Absorption spectra of Au NP colloidal solutions with inset showing a glass vial with colloidal Au NP (S19) solution. **D** Shows a schematic of the setup used for seed-mediated growth of Au NPs

Au NPs were successfully synthesized using the Turkevich method with a solution containing 0.25 mM chloroauric acid (HAuCl₄) and 2.5 mM sodium citrate (Na3Ct). Figures 1A–B show representative S(T)EM images of the Turkevich Au NPs synthesized from varying concentrations of HAuCl₄ of 0.25 and 0.125 mM, respectively, insets showing the size distributions of the particles measured using ImageJ software. The resulting NPs exhibited mean size of 19 ± 2 nm (S19) and 26 ± 3 (S26), which aligns with what is reported by Turkevich et al. and Wuithschick et al. under similar conditions [2, 17]. The mean size here refers to an average diameter calculated by measuring 200 particles using ImageJ and the standard deviation represents the standard deviation of the same size distribution. In the Turkevich method, sodium citrate acts as a reducing agent and a passivating ligand. Polte et al. described the Turkevich method as a seed-mediated growth method in which, during the early stage of the reaction, stable seed particles of diameter around 3 nm are formed. The growth of the Au NP seeds occurs when the remaining gold ions are attached and reduced exclusively in the seed particles' electric double layer (EDL) [18]. The concentration of chloroauric acid has been reported to play a significant role in determining the size of the gold nanoparticles [19]. We employed a higher temperature of 125 °C compared to literature reports which is expected to increase the reaction kinetics, favoring the nucleation and growth processes, ultimately yielding monodisperse Au NPs with the observed characteristics [2, 20].

The polydispersity index (PDI) of the Au NPs measured from dry size remained below 0.01, suggesting a monodisperse population. The optical properties of the colloidal Au NPs were also investigated, and the LSPR maxima showed increasing values of 519 nm and 525 nm, respectively, for increasing particle sizes, consistent with previously reported literature findings. These Au NPs were used as seeds for the seed-mediated growth method discussed below. The unnormalized spectra in Fig. 1C reveal a distinct difference in the concentrations of the Au NP seeds. The concentration of bigger seeds (S26) is significantly lower as compared to smaller Au NPs seeds (S19). This disparity is critical in seed-mediated growth reactions, as the particle number concentration directly dictates the total available surface area for the reduction of HAuCl_4_, significantly influencing the kinetics and efficiency of nanoparticle growth.

### Seed mediated growth of Au NPs via semi-continuous method

The Turkevich method is widely recognized for synthesizing spherical Au NPs between 10 to 30 nm. However, for particles above 30 nm, particle size distribution becomes broader, the sphericity is reduced, and results are less reproducible [21]. To address size constraints, we present a straightforward semi-continuous method for seed-mediated growth of Au NPs. This method leverages pre-prepared Au NP seeds synthesized via the Turkevich method and employs a syringe pump to introduce chloroauric acid under controlled flow rates. Notably, our method offers a distinct advantage: synthesis of desired size Au NPs without the need for surfactants such as CTAB and oleic acid or additional reducing agents such as ascorbic acid, tannic acid, and hydroxylamine [10, 22, 23]. This is achieved by carefully optimizing precursor concentration, flow rate, and temperature. A Design of Experiment (DOE) was devised to comprehensively explore the synthesis parameters, examining the impact of temperature, flow rate, and reducing agent. The table outlining the DOE parameters investigated in this study, along with the representative S(T)EM images of Au NPs resulting from the DOE and their size distributions, are given in the SI.

### Effect of reaction variables

**Fig. 2:**
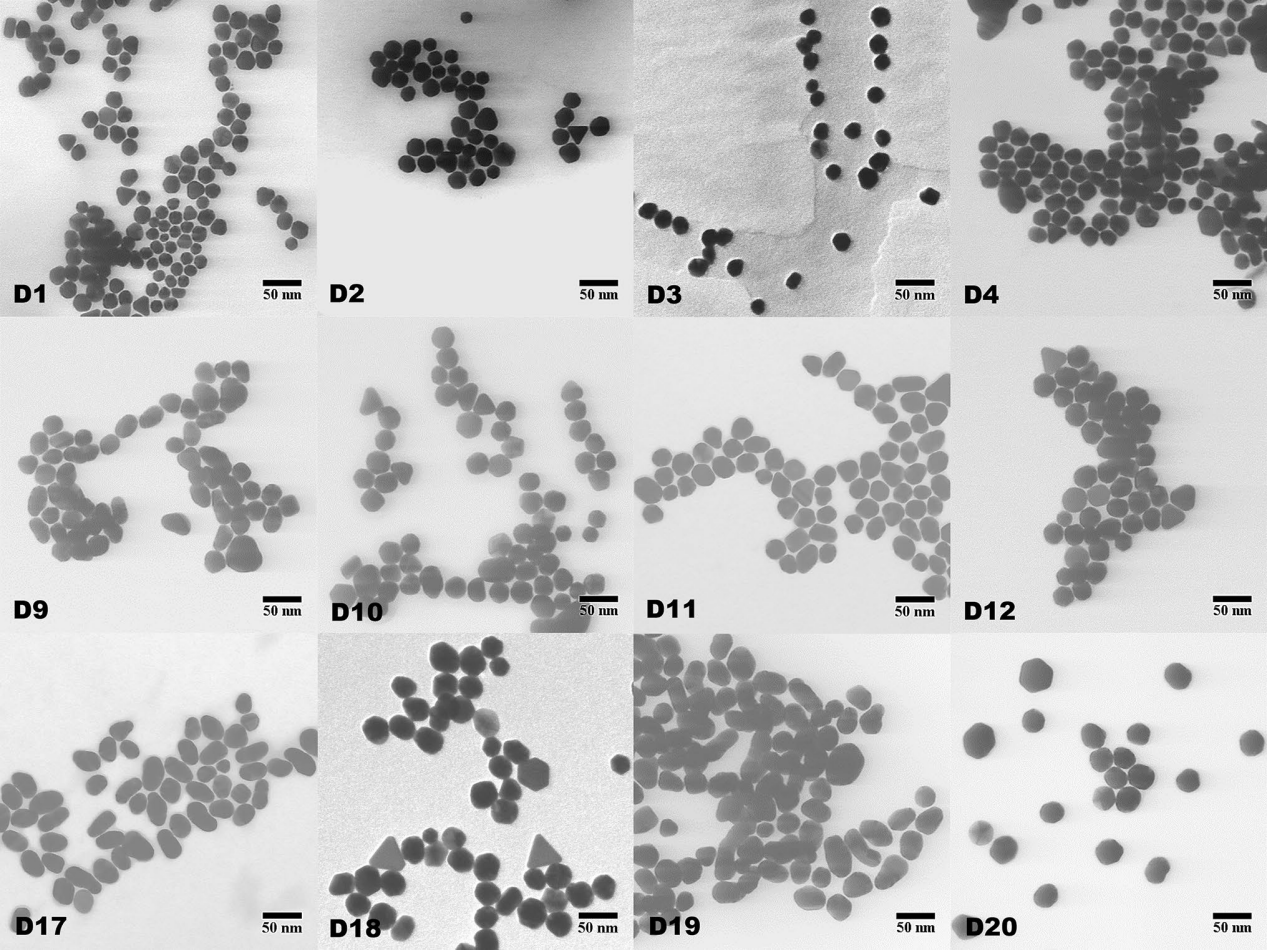
Seeded growth of Au NPs (S19) via a semi-continuous method. S(T)EM images of Au NPs synthesized with (D1-D4) 0.25, (D9-D12) 0.5, and (D17-D20) 1.0 mM HAuCl₄, respectively. On average particle size increases from 22 ± 3 to 27 ± 4 and 32 ± 5 with incrementing HAuCl₄ concentration

**Fig. 3:**
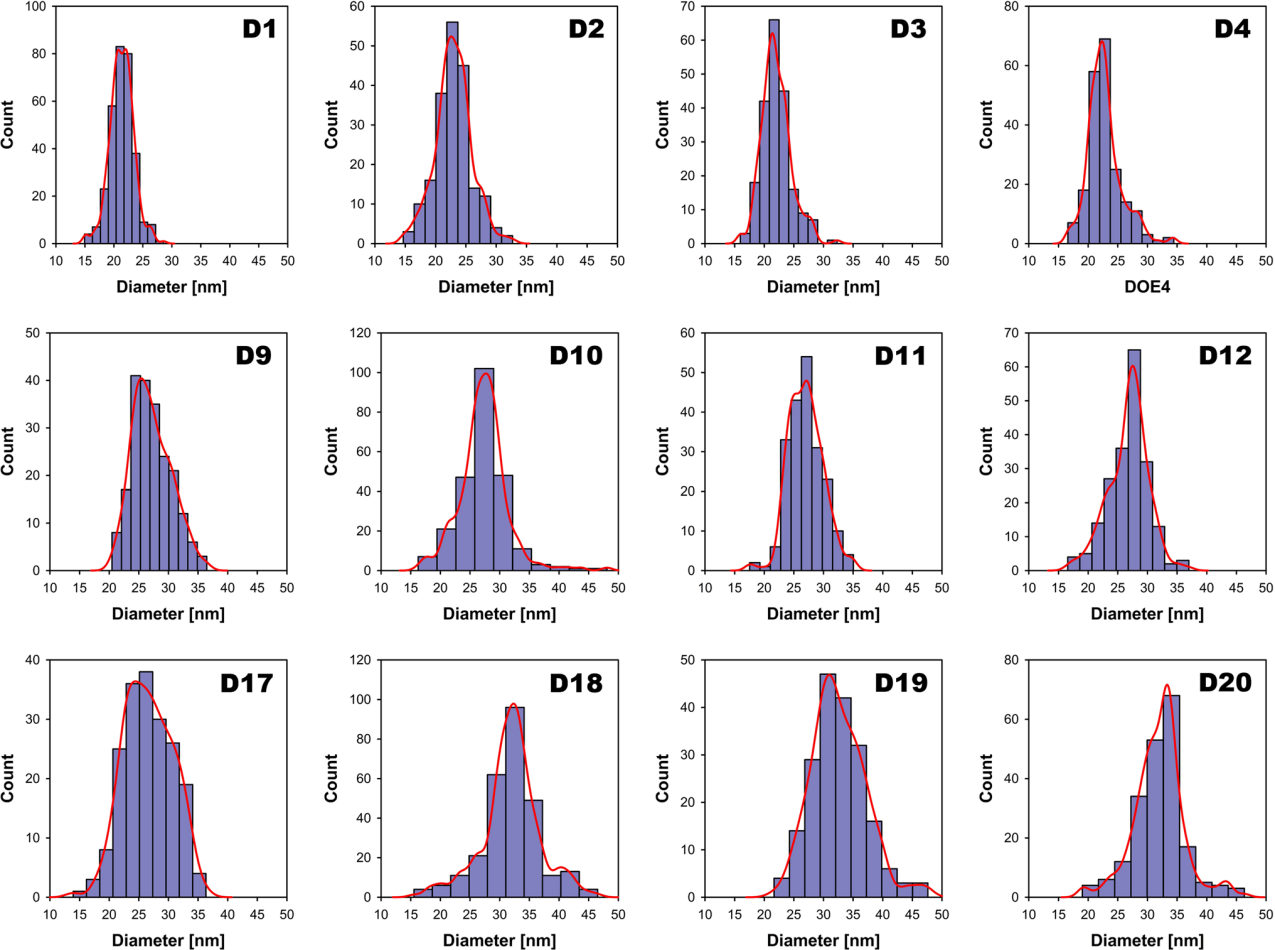
Size distribution analysis of Au NPs (S19) synthesized via a semi-continuous method. Representative histograms with kernel density estimations of Au NP samples (D1–D20) depict size distribution, measured using ImageJ software

Figures 2 (D1-D20) show representative S(T)EM images of Au NPs synthesized via seed-mediated growth of Au NPs at 0.25 (D1-D4), 0.5 (D9-D12), and 1.0 (D17-D20) mM HAuCl₄ concentrations, respectively. Whereas the plotted histograms with kernel density estimation in Fig. 3 (D1-D20) show the size distributions of the particles measured using ImageJ software. On average the dry diameter of Au NPs increased from 22 ± 3 to 27 ± 4 and 32 ± 5 for incrementing concentrations of HAuCl₄. The average particle diameter and standard deviations were calculated by counting at least 200 nanoparticles for each sample. A similar trend for dry diameter was observed across the designed set of experiments. However, variation in shape was observed for DOE 17 and DOE 19 as the synthesized Au NPs exhibited elongated shapes with aspect ratios of ∼1.75. This could be attributed to a lower temperature coupled with a higher gold precursor concentration as both reactions were performed at 70 °C and 1 mM HAuCl₄ concentration.

Based on the morphology of the Au NPs, it is evident that the Au NPs synthesized at 125 °C are mostly spherical. In contrast, those synthesized at 70 °C, especially with higher concentrations of HAuCl₄ (DOE 17, DOE 19), are elongated. This confirms that the concentration of gold chloride and temperature play a crucial role in achieving the desired size and morphology of Au NPs. According to Zeigler et al., it is possible to synthesize spherical Au NPs without boiling the reaction mixture, but the particles may have rough and porous surfaces that tend to merge quickly. In addition, the concentration should be kept below a specific threshold to prevent the formation of polyhedral nanoparticles [13]. Zeigler et al. emphasized the significance of slowly adding the gold precursor to control particle formation and achieve uniform size and shape as a direct injection can lead to variable sizes and morphologies [13].

The shape of Au NPs synthesized through seed-mediated growth can vary significantly with temperature due to differences in the kinetics of atom diffusion and reduction processes. At 125 °C, the higher temperature increases the kinetic energy of gold atoms, which promotes faster and more uniform diffusion across the nanoparticle seeds. This results in a uniform deposition of gold atoms, favoring the formation of spherical nanoparticles because the atoms can effectively rearrange to minimize surface energy and achieve thermodynamic stability. In contrast, at 70 °C, the lower temperature slows down the diffusion and reduction rates. This slower process can lead to anisotropic growth, where certain crystallographic facets grow faster than others, resulting in elongated shapes. The residual sodium citrate from the Turkevich method, acting as both a reducing and stabilizing agent, can further influence this anisotropic growth. At lower temperatures, the interaction between citrate ions and specific facets of the gold seeds can preferentially stabilize certain growth directions, promoting elongation rather than the more uniform growth seen at higher temperatures. Therefore, the key factors contributing to the different shapes are the temperature-dependent kinetics of atom diffusion and reduction and the influence of stabilizing agents on growth direction.

**Fig. 4:**
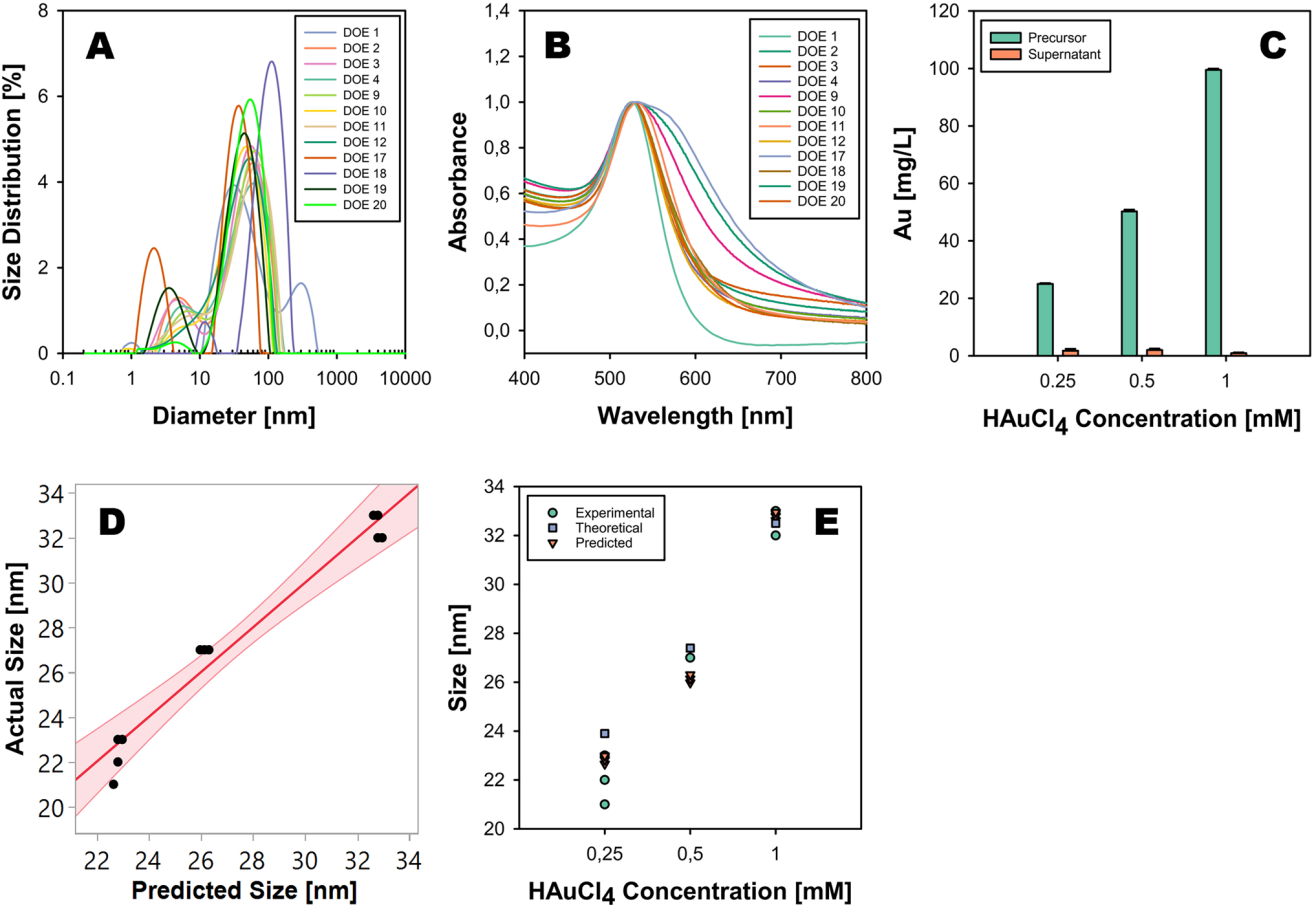
**A** The hydrodynamic size distributions of Au NPs (D1-D20) synthesized via seed-mediated growth method. **B** Absorption spectra of Au NP colloids (D1-D20) obtained from seed-mediated growth of Au NP seeds with 0.25, 0.5, and 1.0 mM HAuCl₄. All spectra are normalized at absorbance 1 for comparison purposes. **C** Shows a graphical representation of initial concentration of gold ions and the amount of unreacted gold leftover in the sample post seed-mediated growth reaction measured with MP-AES. **D** A correlation between the predicted and actual dry size of Au NPs obtained by statistical data analysis using JMP software. **E** Compares experimental, theoretically calculated, and JMP-predicted values of dry size against HAuCl₄ concentration

The hydrodynamic sizes and absorption spectra of the samples (D1–D20) were measured and are presented in Fig. 4A–B. Unlike the dry diameters determined using ImageJ software, the hydrodynamic size distribution does not exhibit a consistent trend. However, in most cases, the intensity weighted size distributions obtained from DLS measurements closely align with the dry size distributions, albeit DLS sizes being larger than S(T)EM sizes. The slightly larger hydrodynamic sizes compared to the dry diameters can be attributed to differences in measurement principles, the DLS sizes representing hydrodynamic diameters of particles in suspension, combined with aggregation both due to interparticle interactions or shelf stability of the dispersions.

In addition, small peaks in the 1–2 nm range were detected, suggesting that the gold nanoparticles (Au NPs) are multifaceted, leading the instrument to detect dimensions within this range. This observation is consistent with the properties of anisotropic nanostructures, even though S(T)EM images do not reveal particles of such dimensions. Samples D17 and D19 exhibit more pronounced peaks in the 1–2 nm range, potentially due to their elongated morphologies contributing to the detected hydrodynamic values.

The absorption spectra, as depicted in Fig. 4B, reveal slight redshifts in the plasmonic peaks. These shifts are subtle overall, with the most notable variations observed in samples DOE 17 and DOE 19, which display a distinct shoulder peak. This shoulder feature corroborates the anisotropic nature of their morphology, providing additional support for their unique structural attributes.

### Statistical analysis

The seed-mediated growth of Au NPs was analyzed using the least squares fit approach using JMP Pro 17 software to understand the significance of reaction variables. The dataset included variables such as HAuCl₄ concentration, precursor flow rate, and temperature, with the response variable being the dry sizes of the synthesized Au NPs.

The results as shown in Fig. 4D indicated a high degree of correlation between the predictors and the response variable, as demonstrated by an R-squared value of 0.962 and a Root Mean Square Error (RMSE) of 1.009. These values suggest that approximately 96% of the variance in the dry sizes of the Au NPs can be explained by the model, demonstrating a strong fit. Additionally, the *p*-value of 0.0001 confirms the statistical significance of the model, aligning well with existing literature where predictive accuracies typically range from 0.80 to 0.90 [24].

The analysis of variance (ANOVA) results showed significant effects of the HAuCl₄ concentration on the dry sizes of Au NPs, with an F-value of 67.39 and a *p-*value of less than 0.0001. However, the precursor flow rate and temperature did not show statistically significant effects under the conditions tested, both having *p-*values of 0.782. This indicates that the concentration of HAuCl₄ significantly affects the size of the Au NPs, whereas the precursor flow rate and temperature do not.

The strong dependency of nanoparticle size on the concentration of HAuCl₄ aligns with the expected mechanism of seed-mediated growth, where the availability of gold ions directly influences the growth rate and final size of the nanoparticles. The insignificant effects of precursor flow rate and temperature within the tested range suggest that, under these specific conditions, the kinetics and thermodynamics of the seed-mediated growth process are predominantly driven by the precursor concentration. These findings provide crucial insights for optimizing the synthesis parameters for Au NPs. By controlling the HAuCl₄ concentration, one can precisely tune the nanoparticle size, while variations in flow rate and temperature have a lesser impact. This could simplify the experimental setup and enhance reproducibility in nanoparticle synthesis. Overall, this study underscores the importance of precursor concentration in the seed-mediated growth of Au NPs and provides a quantitative framework for predicting nanoparticle size based on this critical parameter.

The experimental study utilized a seed-mediated growth method to synthesize Au NPs, employing Au NP seeds with an initial diameter of 19 nm. The particle number of these seeds was calculated using the relationship [25].

1 $${\text{M}} = {\text{N}}_{0} \frac{{\uppi }}{6}{\text{D}}^{3} \times {\uprho }$$

where M is the mass of Au, ${\text{N}}_{0}$ is seed particle number, ${\text{D}}^{3}$ is the diameter of seed particles, and $\uprho$ is the density of gold. This calculated seed number was subsequently used in Eq. 2 to calculate the diameter of Au NPs after growth under varying concentrations of HAuCl₄. Where ${{\text{d}}_{1}}^{3}\text{and }{{\text{d}}_{0}}^{3}$ represent the diameter of Au NPs post-seed-mediated growth and Au NP seeds, respectively [26].

2 $$d_{1}^{3} = d_{0}^{3} + \frac{6 \times M}{{N_{0} \times \pi \times \rho }}$$

$$d_{1}^{3} = \left( {19 \times 10^{ - 7} } \right)^{3} + \frac{{6 \times 1.97 \times 10^{ - 3} }}{{7.1 \times 10^{12} \times 3.14 \times 19.3}}$$

The theoretically calculated diameters using Eq. 2 showed a strong correlation with the experimentally measured diameters, particularly at higher concentrations of HAuCl₄ (0.5 mM and 1 mM). This close agreement suggests that the theoretical model effectively captures the growth dynamics under these conditions, providing a reliable tool for predicting the size of Au NPs in seed-mediated synthesis. The slight deviation observed at the lower concentration of 0.25 mM HAuCl₄, while within the standard error margin, underscores the complexity of nanoparticle synthesis, where slight variations in experimental conditions, such as incomplete reduction of HAuCl₄ or variations in reaction kinetics, can lead to minor discrepancies between theoretical predictions and experimental outcomes.

It is crucial to recognize that the theoretical calculations are based on the assumption of complete reduction of HAuCl₄ during the synthesis of the initial Au NP seeds via the Turkevich method, followed by a uniform growth phase. In reality, factors such as unreacted gold precursors, variations in the reduction kinetics, and the influence of stabilizing agents could introduce subtle variations that manifest as differences between the predicted and observed nanoparticle sizes. MP-AES was employed to assess the conversion efficiency of Au ions into Au NPs. Unreacted Au ions were quantified by centrifuging the reaction mixture, isolating the supernatant, and measuring the Au content. The concentrations of unreacted Au were determined to be 1.8 ± 0.7, 2.0 ± 0.5, and 0.9 ± 0.3 mg/mL for supernatants collected from Au NP samples synthesized with 0.25, 0.5, and 1.0 mM HAuCl₄, respectively. Theoretical calculations indicated that the starting Au concentrations were 24.6, 49.2 and 98.5 mg/L respectively. The conversion efficiencies, represented in Fig. 4C, highlights over 90% conversion of Au ions to Au NPs, underscoring the effectiveness of the seed-mediated growth method in achieving conversion of Au ions into Au NPs. Furthermore, the conversion rates correspond to slight discrepancies between theoretical and experimental particle sizes, with the latter being marginally smaller due to subtle variations in growth kinetics, as shown in Fig. 4E.

Furthermore, the utilization of Eq. 2 provides a valuable framework for tailoring the size of Au NPs in seed-mediated growth systems. By manipulating the concentration of HAuCl₄ and accurately calculating the required gold mass, researchers can precisely control the final nanoparticle diameter, enabling the synthesis of Au NPs with desired size distributions for specific applications in catalysis, biomedical imaging, or sensing technologies.

### Effect of seed size

Our study with 19 nm Au NP seeds demonstrates the critical role of HAuCl₄ concentration in the seed-mediated growth of Au NPs. The results indicate that while temperature does not significantly influence particle size, it is essential for maintaining the sphericity of the Au NPs. This aligns with previous findings that temperature can affect the morphology of nanoparticles by providing the necessary thermal energy for uniform growth [2, 27, 28]. Therefore, to gain deeper insights into the influence of Au NP seeds, we employed a higher flow rate and set the temperature to 125 °C for the seed-mediated growth of 26 nm Au NP seeds. These seeds were synthesized using the Turkevich method with 0.125 mM HAuCl₄ and 2.5 mM sodium citrate dihydrate as the reducing agent. A representative S(T)EM image and the size distribution of 26 nm Au NPs are given in Fig. 1B.

**Fig. 5:**
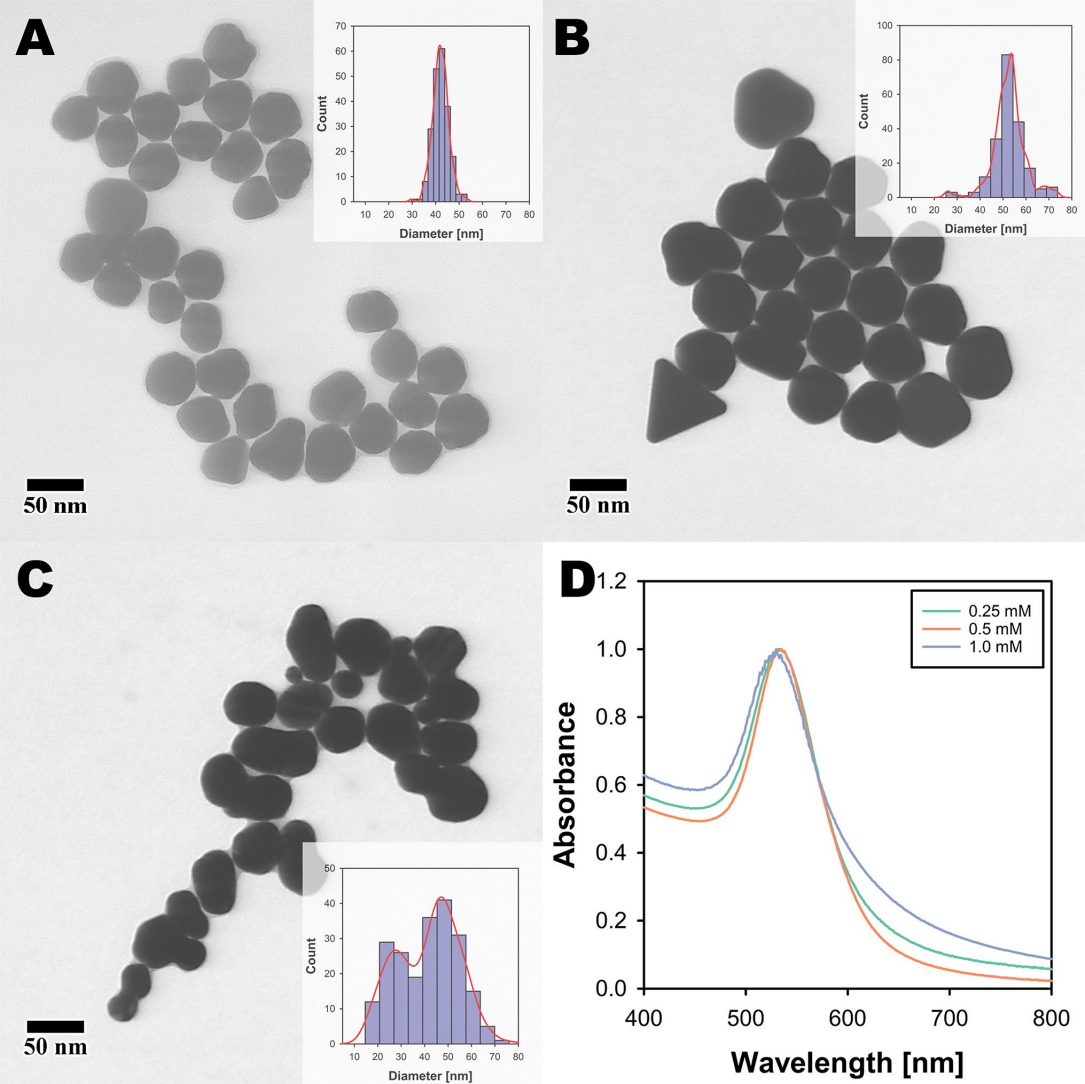
Seeded growth of Au NPs (S26) via a semi-continuous method at 125 °C. S(T)EM images **A**–**C** of Au NPs synthesized with 0.25, 0.5, and 1.0 mM HAuCl₄, respectively, with insets showing particle size distributions measured using Image J. The Au NPs of diameters 42 ± 3, 53 ± 7, and 41 ± 13 were produced with incrementing HAuCl₄ concentration. **D** Absorption spectra of Au NP colloids obtained from seed-mediated growth of Au NP seeds (S26) with 0.25, 0.5, and 1.0 mM HAuCl₄

The Fig. 5A–C shows representative S(T)EM images of Au NPs synthesized via seed-mediated growth of 26 nm Au NP seeds with incrementing concentrations of HAuCl₄ with insets representing corresponding size distributions. Whereas Fig. 1D shows absorption spectra of the colloidal Au NPs. The significant increase in particle size observed at 0.25 and 0.5 mM HAuCl₄ concentrations can be attributed to the availability of more gold ions for reduction, leading to larger particles. However, at 1 mM HAuCl₄, homogeneous nucleation occurs, resulting in a broader size distribution and anisotropic particles. This phenomenon is likely due to high supersaturation, where the high concentration of HAuCl₄, coupled with leftover sodium citrate, exceeds the nucleation threshold, leading to the formation of new nuclei rather than the growth of existing seeds [29]. The ratio of sodium citrate to chloroauric acid, in this case, is 20:1, in contrast to the 19 nm seeds, where the reducing agent to precursor ratio was maintained at 10:1. This higher sodium citrate concentration in the 26 nm seed solution likely results in more unreacted sodium citrate, which could influence the supersaturation levels and subsequently affect the growth dynamics of the seeds.

The observed anisotropy at 1 mM HAuCl₄ suggests that higher precursor concentrations can lead to non-spherical particle growth. This is consistent with the literature, where high precursor concentrations have been shown to induce anisotropic growth due to the differential reduction rates on various crystal facets. The particle sizes measured (42 ± 3 nm, 53 ± 7 nm, and 41 ± 13 nm for 0.25, 0.5, and 1 mM HAuCl₄, respectively) further support the notion that optimal precursor concentration is crucial for achieving uniform particle sizes.

The lower number of seeds prepared from 0.125 mM HAuCl₄ (Fig. 1C) likely resulted in a reduced surface area for Au ion reduction, facilitating nucleation over growth. This is supported by the fact that fewer seeds provide less surface area for the reduction process, leading to the formation of new nuclei when the precursor concentration is high. Additionally, unreacted sodium citrate in larger seeds could contribute to supersaturation, promoting homogeneous nucleation. Additionally, the degree of anisotropy observed can be linked to the initial morphology of the seeds. As depicted in Fig. 1A, B, smaller Au NP seeds tend to exhibit a spherical shape, while larger seeds display multifaceted structures. These morphological characteristics are largely retained, if not slightly enhanced, following the seed-mediated growth process, which aligns with expectations. Although pH variation was not explored within the scope of this study, prior research indicates its significant influence on particle shape. A slightly basic pH (pH ∼8) could be employed during seed synthesis to enhance particle sphericity, potentially offering finer control over particle morphology throughout the growth process [30]. Our findings highlight the delicate balance required in precursor concentration and seed preparation to achieve the desired nanoparticle size and shape.

### Effect of additional citrate

Several methods of seed-mediated growth of Au NPs rely on additional reducing agents for particle growth and stability. Bastus et al. proposed sodium citrate-assisted seed-mediated growth of Au NPs [12]. Similarly, Zeigler et al. reported a method that uses trisodium citrate and ascorbic acid as reducing agents [13]. Brown et al.’s method relies on hydroxylamine as a reducing agent for HAuCl₄ [23]. Even though we demonstrate a method without additional reducing agents, we wanted to explore if additional sodium citrate had any impact on the size and morphology of Au NPs. For this purpose, a specific amount of sodium citrate dihydrate was added to the Au NP seed solution in addition to any leftover citrate from the Turkevich reaction, which was used to synthesize Au NP seeds. The seed-mediated growth of Au NPs with additional sodium citrate dihydrate was done with 0.5 mM of HAuCl₄. Representative S(T)EM images of Au NPs synthesized with increasing citrate concentrations are shown in Fig. 4A–D, with insets displaying particle size distributions measured using ImageJ. A bar chart showing dry particle size at varying amounts of citrate (E) and normalized UV–Vis spectra of Au NP colloids (F) are also included.

**Fig. 6:**
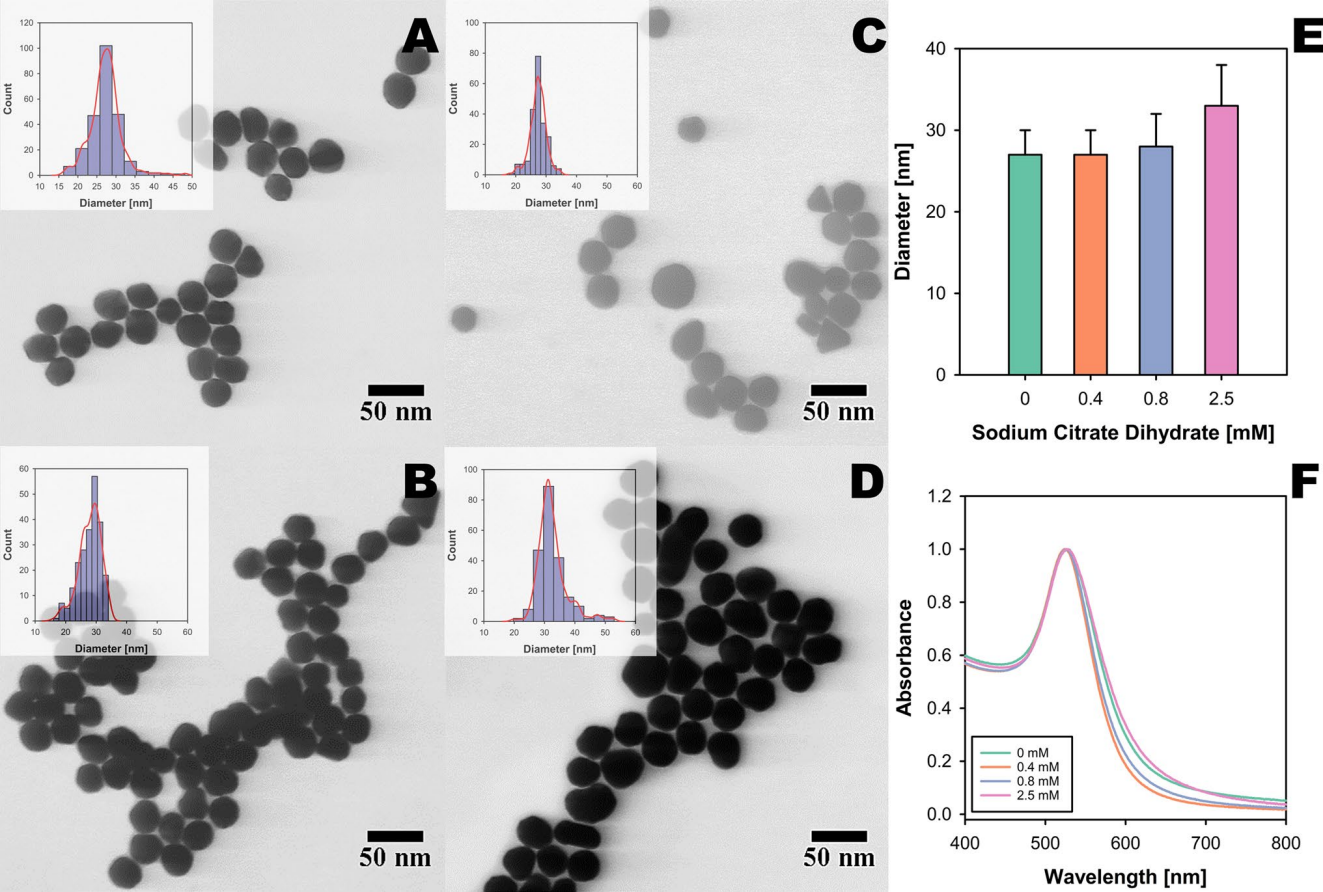
Effect of variable amounts of citrate on particle size and morphology. **A**–**D** S(T)EM images of Au NPs post-seed-mediated growth with additional citrate concentrations of 0, 0.4, 0.8, and 2.5 mM, respectively. **E** Effect of additional citrate on particle diameter. **F** Absorption spectra of Au NPs obtained from seed-mediated growth of Au NP seeds at varying concentrations of additional citrate. All spectra are normalized at absorbance 1 for comparison purposes

It was observed that the dry size and LSPR (Fig. 6E) remained consistent within a range of 0–0.8 mM of additional sodium citrate. This suggests that within this range of citrate concentration, the size of the Au NPs is not significantly affected. It could be assumed that the citrate ions at these concentrations are insufficient to impact the reduction rate of the gold ions notably, and hence, the size of the resulting nanoparticles. Furthermore, HAuCl₄ could be the rate-limiting factor under these conditions, and a higher concentration of HAuCl₄ might be needed to induce a significant impact on size. However, it should be noted that a higher concentration of gold precursor coupled with a higher concentration of reducing agent could overcome the threshold for supersaturation to favor nucleation of new Au NPs leading to a polydisperse population. Relatively narrow plasmonics of Au NPs suggest the stability of Au NPs referring to the presence of sufficient amounts of sodium citrate to stabilize particles post-synthesis.

A slightly bigger increase in the dry size of Au NPs was observed for a citrate concentration of 2.5 mM, reaching a dry size of 33 ± 5 nm. This increase suggests that the rate of reduction of gold ions is significantly enhanced at higher citrate concentrations, leading to the formation of larger nanoparticles. However, this led to a higher degree of anisotropy among the Au NPs along with a wider size distribution. This is further confirmed by a slight increase in the width of the LSPR for Au NPs synthesized with 2.5 mM of additional citrate.

### Kinetics study

**Fig. 7:**
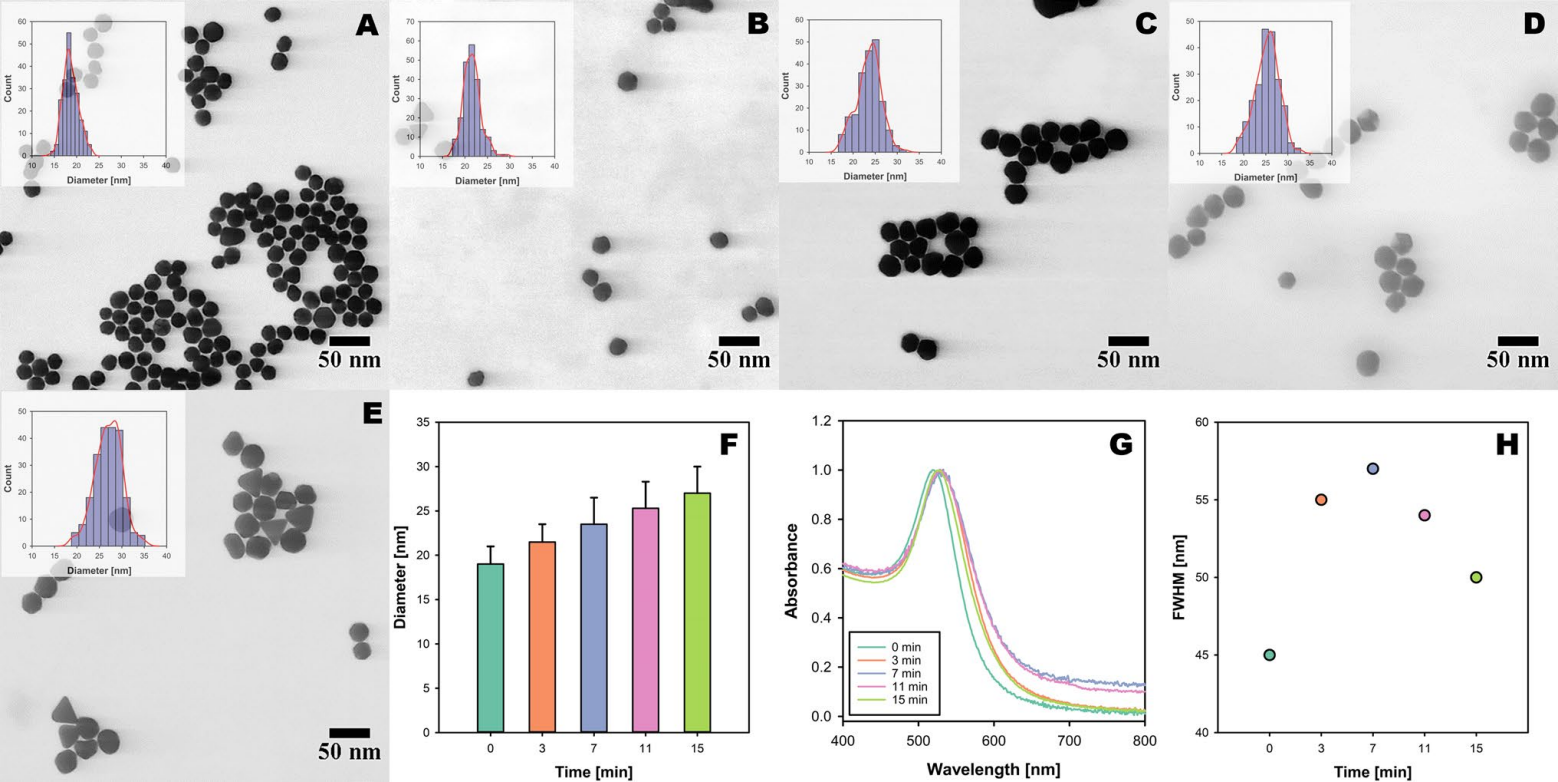
Monitoring the growth of Au NPs over regular intervals from 0 to 15 min. **A**–**E** S(T)EM images of Au NPs at 0, 3, 7, 11, and 15 min, respectively. **F** Increase in the diameter of the Au NPs over time. **G** Normalized absorbance spectra of Au NP colloids from kinetics study. **H** FWHM of Au NPs from kinetics study measured from UV–Vis plots

To gain insights into how the seeds grow as a function of time, we performed a kinetics study, in which small aliquots of samples from the reaction mixture were taken at 3-, 7-, 11-, and 15-min intervals and analyzed with S(T)EM and UV–Vis shown in Fig. 7A–H. The size of Au NP seeds increased from 19 ± 2 nm to 21.5 ± 2 (3 min), 23.5 ± 3 (7 min), 25.3 ± 3 (11 min), and 27 ± 3 (15 min). This gradual increase in Au NP size indicates that a continuous reduction of ${\text{Au}}^{3+}$ ions to ${\text{Au}}^{0}$ atoms resulted in the direct incorporation of ${\text{Au}}^{0}$ to available Au NP seeds. From the available data points, we observed uniform and continuous growth of Au NPs throughout the reaction period. The S(T)EM images do not show any smaller Au NPs suggesting an absence of primary nucleation. This could be due to the slow addition of HAuCl_4_ and a limited amount of citrate available, which resulted in supersaturation to stay under the desired conditions for growth to be favored. One probable hypothesis to explain these results could be that the continuous growth of Au NPs would occur upon commencing the addition of gold precursor due to the high affinity of gold atoms for the gold seeds and the significantly lower energy barrier required for heterogeneous nucleation [31].

The plasmon peaks for kinetics data saw their redshift from 519 nm for Au NP seeds to 529 nm (3, 7 min), 528 nm (11 min), and 526 nm for the final data point at 15 min. The initial redshift and broadening of the LSPR peaks can be attributed to both the growth and the aggregation of Au NPs. This observation is supported by the increased full-width half maximum (FWHM) values at earlier time points, indicating a wider size distribution or aggregation of particles. However, over time, the observed blueshift in the LSPR peaks, despite the increase in nanoparticle size, may be due to a decrease in the extent of aggregation. As the particles become more separated and well-defined, the plasmon resonance shifts to shorter wavelengths, resulting in narrower and blue-shifted peaks. The initial increase in FWHM suggests a broad size distribution or aggregation, but as the reaction progresses, the FWHM decreases, indicating a transition to a more uniform size distribution of Au NPs. This trend further confirms the initial aggregation and subsequent refinement of particle size and distribution during the reaction.

## Conclusion

In this study, we report a simple approach to synthesizing spherical Au NPs with a controlled size using a one-step seed-mediated growth method. This method uses pre-synthesized Au NP seeds via the Turkevich method and grows them to a desirable size, overcoming the size constraints of the Turkevich method. The key to this method is carefully adjusting various factors such as the concentration of the gold precursor, seed solution, reducing and capping agent, and temperature. The concentration of HAuCl₄ proved to be the most significant variable responsible for size variation whereas, the sphericity of Au NPs showed a temperature dependency. This method can synthesize Au NPs of up to 53 nm diameter in a single growth step. The ability of this method to synthesize citrate stabilized, water soluble Au NPs, with sizes ranging between 21 and 53 nm, and a controlled concentration, expands their widespread use in biology and medicine. These nanoparticles can be further modified with various biologically active molecules, such as deoxyribonucleic acid (DNA), peptides, and antibodies, making them suitable candidates for biosensing and drug delivery.

## Supplementary Information

Additional file1 (DOCX 3757 KB)

## Data Availability

All data generated or analyzed during this study are included in this published article and its supplementary information files.
