# Supplementary material for: Controlled growth of citrate-stabilized gold nanoparticles using a semi-continuous seed-mediated route
Source: Discov Nano. 2025 Feb 17;20(1):39. doi: 10.1186/s11671-025-04189-8 (PMC11833013; doi:10.1186/s11671-025-04189-8)
Supplement: Supplementary file 1 — Additional file1 (DOCX 3757 KB) [file 11671_2025_4189_MOESM1_ESM.docx]

Supporting Information

***Controlled Growth of Citrate-Stabilized Gold Nanoparticles Using A Semi-Continuous Seed-Mediated Route***

Muhammad Bilal, Sulalit Bandyopadhyay*

Particle Engineering Centre, Department of Chemical Engineering, Norwegian University of Science and Technology, Trondheim, 7491, Norway.

Email: [sulalit.bandyopadhyay@ntnu.no](mailto:sulalit.bandyopadhyay@ntnu.no)

# Design of Study

The supporting information provides comprehensive data on the size-controlled synthesis of gold nanoparticles (Au NPs) via the seed-mediated growth method. Table 1 summarizes the design of experiments (DOE), detailing the effects of varying HAuCl₄ concentrations, precursor flow rates, and temperatures on the dry size, hydrodynamic size, and PDI (%) of the synthesized Au NPs. Table 2 presents an overview of various seed-mediated growth methods from the literature, outlining the reducing agents and surfactants/stabilizing agents used, along with the maximum nanoparticle sizes achieved, the size increments per step, and noted challenges such as low yield or the need for multiple growth steps.

| **Sr. No.** | **HAuCl4**  **Conc. [mM]** | **Seed Size [nm]** | **Precursor Flowrate [µL/min]** | **Temperature [****°C]** | **DOE No.** | **Dry Size [nm]** | $\mathbf{D}_{\mathbf{h}}$ **Size**  **[nm]** | **PDI (%)** |
| --- | --- | --- | --- | --- | --- | --- | --- | --- |
| 1 | 0.25 | 19 | 670 | 70 | DOE 1 | 21 ± 2 | 45 ± 7 | 26 ± 2 |
| 2 | 0.25 | 19 | 670 | 125 | DOE 2 | 23 ± 3 | 31 ± 1 | 30 ± 2 |
| 3 | 0.25 | 19 | 335 | 70 | DOE 3 | 22 ± 2 | 31 ± 2 | 30 ± 1 |
| 4 | 0.25 | 19 | 335 | 125 | DOE 4 | 23 ± 3 | 36 ± 2 | 27 ± 3 |
| 5 | 0.5 | 19 | 670 | 70 | DOE 9 | 27 ± 3 | 38 ± 1 | 28 ± 1 |
| 6 | 0.5 | 19 | 670 | 125 | DOE 10 | 27 ± 4 | 33 ± 1 | 28 ± 1 |
| 7 | 0.5 | 19 | 335 | 70 | DOE 11 | 27 ± 3 | 39 ± 1 | 27 ± 2 |
| 8 | 0.5 | 19 | 335 | 125 | DOE 12 | 27 ± 3 | 31 ± 1 | 28 ± 1 |
| 9 | 1 | 19 | 670 | 70 | DOE 17 | 33 ±6 | 21 ± 2 | 31 ± 3 |
| 10 | 1 | 19 | 670 | 125 | DOE 18 | 32 ± 5 | 90 ± 2 | 25 ± 2 |
| 11 | 1 | 19 | 335 | 70 | DOE 19 | 33 ± 5 | 26 ± 1 | 30 ±2 |
| 12 | 1 | 19 | 335 | 125 | DOE 20 | 32 ± 4 | 42 ± 1 | 24 ± 4 |

**Table 1:** Design of Experiments (DOE) showing the effect of HAuCl₄ concentration, precursor flow rate, and temperature on the dry and hydrodynamic ($D_{h}$) diameters of gold nanoparticles (Au NPs).

# Effect of Temperature

Figure 1 presents representative S(T)EM images of Au NPs synthesized at 70°C, with all other parameters, including precursor concentration, seed size, and flow rates, held constant as in the S(T)EM images shown in Figure 4 of the main text. This figure highlights its influence on particle morphology and size distribution by isolating temperature as the variable. At elevated temperatures, the growth rate is more uniform due to enhanced diffusion of precursor ions around the seeds. This reduces the likelihood of anisotropic growth or defects that result in random shapes [1]. Furthermore, at higher temperatures, gold atoms can rearrange more freely, favoring configurations with lower surface energies. This leads to the formation of thermodynamically stable spherical shapes. Low temperatures often result in kinetic control of growth, where particles grow according to local conditions, leading to nonuniform shapes. At high temperatures, thermodynamic control is dominant, producing uniform particles [2, 3].


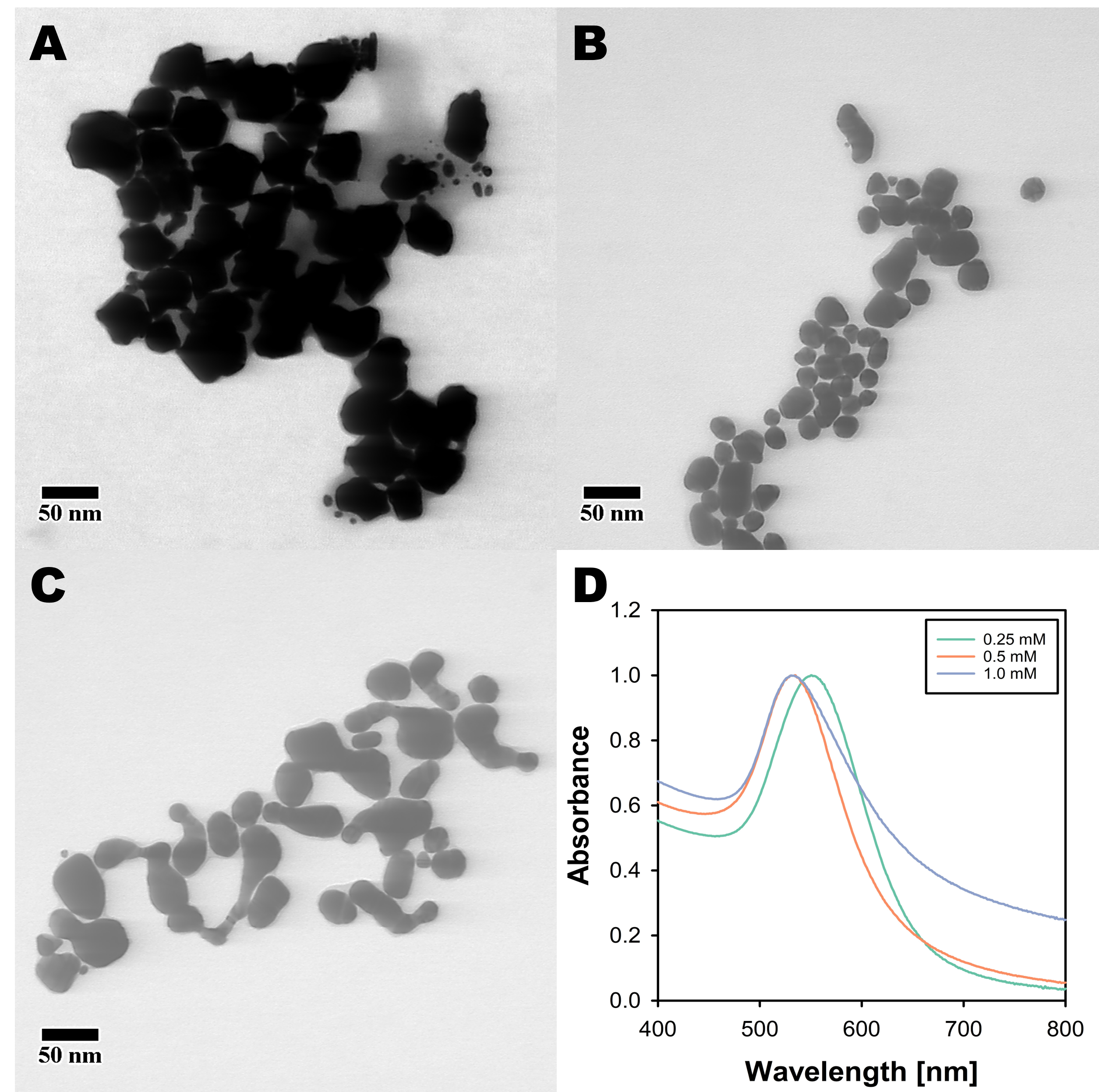


**Figure 1.** Seeded growth of Au NPs (S26) via a semi-continuous method at 70°C. S(T)EM images (A-C) of Au NPs synthesized with 0.25, 0.5, and 1.0 mM ${HAuCl}_{4}$, respectively. (D) Absorption spectra of Au NP colloids obtained from seed-mediated growth of Au NP seeds (S26) with 0.25, 0.5, and 1.0 mM ${HAuCl}_{4}$. The scale bar is 50 nm.

| **Reducing Agent** | **Surfactant/ Stabilizing Agent** | **Max Size Obtained (nm)** | **Max Size Increase in a Single-Step (nm)** | **Growth Method** | **Shortcomings** | **Reference** |
| --- | --- | --- | --- | --- | --- | --- |
| Sodium Citrate | Sodium Citrate | 180 | 30 | Multiple growth steps | 14 growth steps required | [4] |
| Sodium Citrate, Ascorbic Acid | Sodium Citrate | 294 | 144 | Multiple growth steps | Low yield | [5] |
| Ascorbic Acid | CTAB, PVP | 37 | 33 | Multiple growth steps | Multiple growth steps, anisotropy, use of surfactant | [6] |
| Ascorbic Acid | CTAB | 153 | 135 | One-step growth | Low yield,  Surfactant | [7] |
| Sodium Citrate, Hydroquinone | Sodium Citrate | 93 | 80 | One-step growth | Low yield (25 µL seeds),  Nucleation leading to 3 nm Au NPs | [8] |

**Table 2:** Overview of various growth methods for synthesizing Au NPs using different reducing agents and surfactants or stabilizing agents.

# References

1. Zhao, X., et al., *Mechanistic Study of Seed-Mediated Growth of Gold Rhombic Dodecahedra.* The Journal of Physical Chemistry C, 2021. **125**(49): p. 27394-27402.

2. Wu, F. and Q. Yang, *Ammonium bicarbonate reduction route to uniform gold nanoparticles and their applications in catalysis and surface-enhanced Raman scattering.* Nano Research, 2011. **4**(9): p. 861-869.

3. Liu, F.-K., et al., *Microwave Heating for the Preparation of Nanometer Gold Particles.* Japanese Journal of Applied Physics, 2003. **42**(6S): p. 4152.

4. Bastús, N.G., J. Comenge, and V. Puntes, *Kinetically controlled seeded growth synthesis of citrate-stabilized gold nanoparticles of up to 200 nm: size focusing versus Ostwald ripening.* Langmuir, 2011. **27**(17): p. 11098-11105.

5. Ziegler, C. and A. Eychmuller, *Seeded growth synthesis of uniform gold nanoparticles with diameters of 15− 300 nm.* The Journal of Physical Chemistry C, 2011. **115**(11): p. 4502-4506.

6. Jana, N.R., L. Gearheart, and C.J. Murphy, *Seeding growth for size control of 5− 40 nm diameter gold nanoparticles.* Langmuir, 2001. **17**(22): p. 6782-6786.

7. Huang, Y. and D.-H. Kim, *Synthesis and Self-Assembly of Highly Monodispersed Quasispherical Gold Nanoparticles.* Langmuir, 2011. **27**(22): p. 13861-13867.

8. Khoury, R.A., et al., *Monitoring the Seed-Mediated Growth of Gold Nanoparticles Using in Situ Second Harmonic Generation and Extinction Spectroscopy.* The Journal of Physical Chemistry C, 2018. **122**(42): p. 24400-24406.
